# Longitudinal trajectories of non-suicidal self-injury, suicidal ideation, and suicide attempts following standardized outpatient emergency management in adolescents

**DOI:** 10.1186/s13034-026-01204-3

**Published:** 2026-11-06

**Authors:** Irina Jarvers, Angelika Ecker, Daniel Schleicher, Alexandra Otto, Romuald Brunner, Stephanie Kandsperger

**Affiliations:** Department of Child and Adolescent Psychiatry and Psychotherapy, University of Regensburg, Regensburg, Germany

**Keywords:** Adolescents, Emergency, Non-suicidal self-injury, Suicidality, Safety plan

## Abstract

**Background:**

Emergency presentations for non-suicidal self-injury (NSSI), suicidal ideation, and suicide attempts in youth have increased markedly. Behavioural and ideational components of self-injury and suicidality may follow partially dissociable courses, underscoring the need for longitudinal evaluation after emergency care.

**Objectives:**

To examine longitudinal changes in NSSI behaviour, NSSI thoughts, suicidal ideation, and suicide attempts after standardized outpatient emergency management; identify psychological and familial predictors; and assess the perceived benefit of safety planning.

**Methods:**

Participants were 105 adolescents (mean age = 14.9 years) presenting in acute crisis to an outpatient emergency service with NSSI and/or suicidal ideation/suicide attempt, followed up twice. Measures included NSSI and suicidal thoughts and behaviour, emotional reactivity, psychological distress, patient and parental motivation, parental stress, and perceived benefit of the safety plan. Changes over time were analysed using non-parametric tests of both frequency and prevalence, and predictors using linear mixed-effects models.

**Results:**

Changes differed by dimension: suicide attempts declined in both frequency and prevalence, NSSI behaviour declined in frequency, and NSSI thoughts and suicidal ideation in prevalence while overall frequency was unchanged. Emotional reactivity, general psychological distress, and patient motivation improved. Higher patient motivation was independently associated with lower NSSI behaviour over time, while no variables significantly predicted NSSI thoughts. Parental, but not adolescent, perceived benefit of the safety plan was associated with reduced NSSI behaviour at follow-up.

**Conclusions:**

Following emergency management, self-injurious behaviour and ideation followed partially dissociable courses. Behavioural improvement was linked to adolescent motivation and parental engagement, whereas ideation improved less completely: fewer adolescents reported suicidal or NSSI-related thoughts, but their frequency did not decline. Ideation therefore warrants continued, targeted monitoring even when behaviour improves.

**Supplementary Information:**

The online version contains supplementary material available at https://doi.org/10.1186/s13034-026-01204-3.

## Introduction

Mental health emergencies involving children and adolescents have increased substantially over the past decade and represent a major challenge for emergency and outpatient psychiatric services [1–5]. Emergency presentations frequently constitute the first point of contact with mental health care and play a crucial role in shaping longer-term treatment trajectories, making early identification and timely intervention particularly important [1, 2]. Non-suicidal self-injury (NSSI) and suicidal ideation account for a large proportion of psychiatric emergencies in youth [6, 7] and are highly prevalent in adolescence; epidemiological data from 11 European countries indicate a lifetime prevalence of direct self-injurious behaviour of 27.6%, with nearly 8% reporting repetitive behaviour [8]. Self-injurious thoughts and behaviours (SITB) encompass a spectrum ranging from non-suicidal thoughts and acts to suicidal ideation and attempts involving intent to die [9]. Although NSSI is, by definition, carried out without intent to die, it is strongly associated with suicidal ideation and behaviour [10]. Longitudinal analyses indicate that NSSI and suicidal ideation are dynamically and reciprocally related over time at the within-person level, with emotion-related mechanisms such as irritability contributing to their bidirectional association [11]. At the same time, trajectory-based studies demonstrate substantial heterogeneity in the co-development of NSSI and suicidal ideation, with distinct subgroups showing differing levels and directions of change across adolescence [12]. Importantly, clinical longitudinal studies further suggest that behavioural and cognitive components of SITB may follow partially dissociable courses, such that cessation of NSSI behaviour does not necessarily entail normalization of suicidal ideation, particularly in clinically referred adolescents [13, 14]. Cessation of direct self-injurious behaviour (regardless of suicidal intent) is nonetheless associated with a reduced subsequent risk of suicidal ideation and behaviour [13], making early behavioural reduction a critical intervention target in emergency care. Accordingly, safety planning interventions are widely implemented as brief, structured approaches aimed at promoting behavioural stabilization. These interventions typically focus on strengthening coping strategies, restricting access to means, and facilitating help-seeking [15]. However, empirical evidence regarding their specific impact on NSSI behaviour and suicidal ideation in adolescents remains limited [16]. Moreover, prior work in adolescents presenting for emergency care has identified emotional reactivity and motivational factors as central correlates of self-injurious behaviour, and highlighted the importance of parental involvement during crisis intervention [17–19]. However, little is known about whether these psychological and familial factors predict longitudinal change following emergency intervention. Given prior evidence that behavioural and ideational components of SITB may follow partially dissociable trajectories, the present analyses examine NSSI and suicidal outcomes both jointly and as distinct clinical endpoints. The present study examined longitudinal changes in NSSI behaviour, NSSI thoughts, suicidal ideation, and suicide attempts following standardized emergency management, investigated psychological and familial predictors of clinical course, and explored the perceived benefit of safety planning.

## Methods

### Study design and setting

This prospective longitudinal observational study was conducted at the emergency outpatient department of the Clinic of Child and Adolescent Psychiatry, Psychosomatics and Psychotherapy of the University Regensburg, Germany, which provides rapid diagnostic assessment and early intervention for acute psychiatric crises, including SITB. Assessments were conducted across two structured clinical appointments following emergency presentation, with two subsequent follow-up assessments.

### Participants

Participants were children and adolescents aged 11–18 years who presented for rapid crisis assessment at the outpatient emergency service with current or recent SITB (including suicidal ideation or attempts). Inclusion criteria were the presence of SITB and sufficient language proficiency to complete self-report measures. Exclusion criteria included severe cognitive impairment, acute psychotic symptoms, prolonged inpatient treatment (> 12 days), or ongoing regular outpatient treatment by established child and adolescent psychiatric services that did not require a specified treatment offer. Adolescents meeting this criterion continued their existing treatment and were not offered the additional appointments (T2 and T3). Adolescents assessed at T1 who required prolonged inpatient treatment (more than 12 nights) received acute inpatient care accordingly and were excluded from the present study. The cohort therefore comprises adolescents whose acute crisis was considered manageable on an outpatient basis. Recruitment took place between July 2019 and June 2021. Participants who discontinued participation or did not complete follow-up assessments were excluded from longitudinal analyses.

### Procedure

**Fig. 1:**
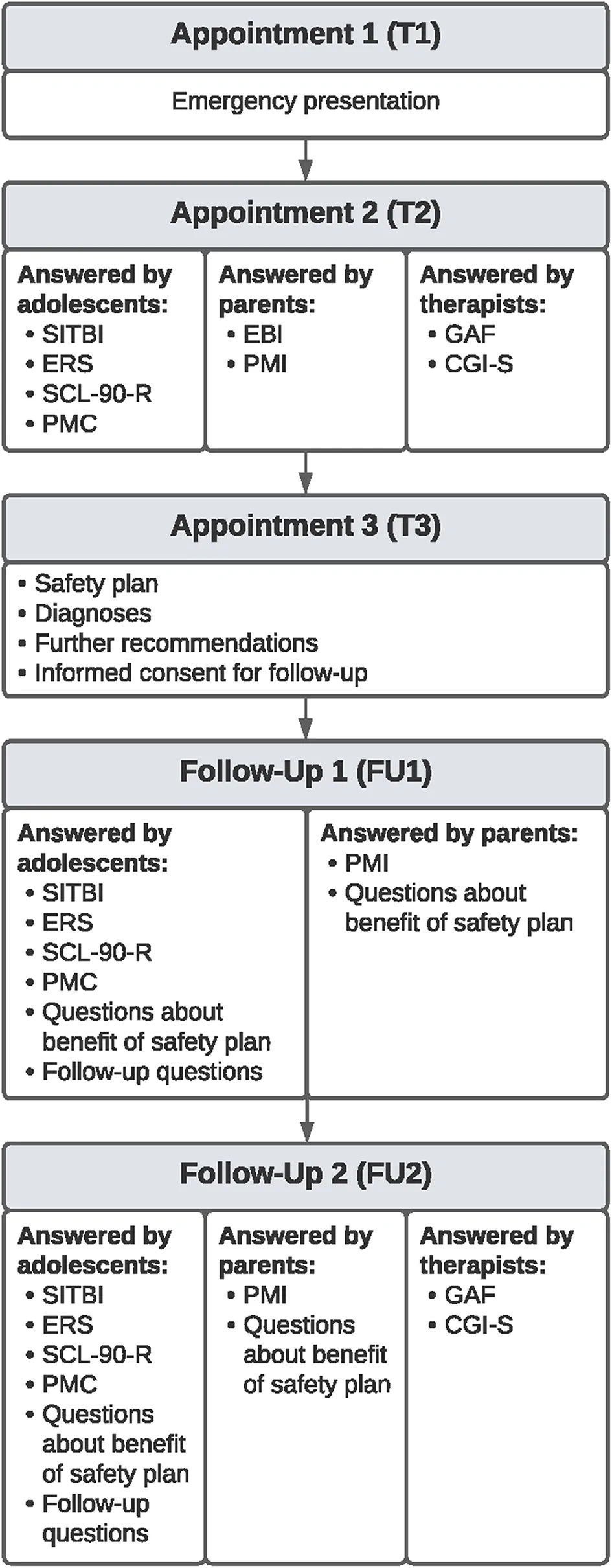
Overview of study appointments and measures. Note. SITBI = Self-Injurious Thoughts and Behaviors Interview; ERS = Emotional Reactivity Scale; SCL-90-R = Symptom Checklist–90–Revised; PMC = Patient Motivation for Change; EBI = Eltern-Belastungs-Inventar (Parenting Stress Index); PMI = Parental Motivation Inventory; GAF = Global Assessment of Functioning; CGI-S = Clinical Global Impressions–Severity scale. T2 = structured clinical appointment, FU1 = first follow up, FU2 = second follow up

In the present study, standardized emergency management refers not to a single intervention delivered at the point of emergency presentation, but to a structured rapid-response pathway: following an acute emergency presentation, adolescents received immediate crisis care and were then offered a fixed sequence of promptly scheduled appointments comprising systematic multi-informant characterization and a collaboratively developed safety plan. This pathway is distinct from usual care, in which structured follow-up appointments are typically not provided after an emergency presentation. Following emergency presentation (T1), participants entered this standardized protocol, comprising two subsequent structured clinical appointments (T2 and T3) and two follow-up assessments (FU1 and FU 2) (see Fig. 1). At the emergency presentation (T1), adolescents received acute clinical crisis care as usual, including de-escalation and assessment of acute symptomatology, immediate treatment needs, and immediate safety. In contrast to usual care, in which such contacts typically conclude without structured follow-up, participants were scheduled for a standardized sequence of promptly arranged appointments (T2 and T3). This offer was made to all eligible adolescents, with the exception of those who were already receiving regular outpatient treatment from established child and adolescent psychiatric services and who did not require a specified treatment offer.

At the second appointment (T2), adolescents underwent comprehensive diagnostic evaluation, including structured clinical interviews and self-report assessments of SITB, emotional reactivity, general psychological distress, and motivation to refrain from self-injury. Parents completed measures of parental stress and motivation, and clinicians rated global functioning and overall illness severity. T2 served as the baseline assessment for the present analyses, as this was the first time point at which complete multi-informant data (adolescent, parent, and clinician) were available.

At the third appointment (T3), diagnoses were finalized and reported to legal guardians, treatment recommendations were provided, and an individualized safety plan was collaboratively developed with the adolescent and, where appropriate, their parents or guardians. Written informed consent for longitudinal follow-up was obtained at this stage. All three appointments (T1, T2, and T3) were conducted in person at the outpatient department.

Follow-up assessments were conducted at two subsequent time points (FU1 and FU2). At both follow-ups, adolescents completed self-report assessments of SITB, emotional reactivity, general psychological distress, motivation, and perceived benefit of the safety plan. Parents completed measures of parental motivation and rated the perceived benefit of the safety plan. At FU2, clinicians again rated global functioning and illness severity. Participants received a €25 voucher upon completion of FU2 as compensation for their time. Follow-up assessments were conducted in person where possible; owing to COVID-19 restrictions during the recruitment period, approximately 40% of follow-up interviews were conducted by telephone.

The interval between T1 and T2 was *M* = 8.04 days (*SD* = 5.90), and between T1 and T3 *M* = 18.37 days (*SD* = 9.71). Follow-up assessments were conducted approximately four weeks (FU1: *M* = 31.97 days, *SD* = 6.34) and eight weeks (FU2: *M* = 60.56 days, *SD* = 7.16) after completion of the emergency management (T3).

### Measures

#### Self-injurious thoughts and behaviours

Self-injurious thoughts and behaviours were assessed using the long version of the Self-Injurious Thoughts and Behaviors Interview (SITBI; [20]), a semi-structured interview assessing the presence, frequency, and characteristics of NSSI behaviour, NSSI thoughts, suicidal ideation, and suicide attempts. The full interview was administered; for the present analyses, only the sections assessing NSSI thoughts and behaviour and suicidal ideation and attempts were used. In addition to frequency measures, adolescents rated the intensity of SITB and suicidal thoughts and attempts at their worst time point. The SITBI demonstrates strong interrater reliability and convergent validity in adolescent samples [21].

#### Emotional reactivity

Emotional reactivity was assessed using the German version of the Emotional Reactivity Scale (ERS; [17, 22]), a self-report questionnaire measuring emotional sensitivity, emotional intensity, and emotional persistence. The ERS has shown good internal consistency and construct validity in adolescent and clinical samples [22]. In the present sample, internal consistency was good to excellent (total α = 0.95; sensitivity α = 0.91, intensity α = 0.89, persistence α = 0.76).

#### General psychological distress

General psychological distress was measured using the Symptom Checklist–90–Revised (SCL-90-R; [23]; German version: [24]). The Global Severity Index (GSI) can be computed from the SCL-90-R and was used as an indicator of overall symptom burden. In the present sample, internal consistency was excellent (α = 0.97). The GSI has demonstrated excellent internal consistency and good construct validity in clinical samples [24].

#### Patient motivation for change

Patients’ motivation to stop non-suicidal self-injury was assessed using a 6-item self-report questionnaire adapted from brief intention and self-efficacy measures originally developed in the context of health behaviour change and emergency self-harm interventions [25, 26]. The Patient Motivation for Change (PMC) questionnaire comprises two subscales assessing behavioural intention to refrain from self-injury (3 items) and self-efficacy regarding the ability to stop self-injury (3 items). Items are rated on a 7-point Likert scale, with higher scores indicating greater motivation. Subscale scores were computed as sum scores. In the present sample, internal consistency was good to excellent across assessment points (behavioural intention: α = 0.87–0.89; self-efficacy: α = 0.91–0.93).

#### Parental motivation

Parental motivation for treatment was assessed using the German version of the Parental Motivation Inventory (PMI; [27]), a parent-report measure assessing parents’ readiness to engage in their child’s treatment. The PMI consists of Likert-type items assessing treatment commitment and perceived importance of change. The PMI has demonstrated good internal consistency and predictive validity in outpatient child and adolescent psychiatric samples [27]. In the present sample, internal consistency was good (α = 0.87).

#### Parental stress

Parental stress was assessed using the Eltern-Belastungs-Inventar (EBI; [28]), a validated German translation of the Parenting Stress Index (PSI [29]). The EBI measures stress related to parenting, child characteristics, and parent–child interaction. The EBI assess parental stress on three domains, the child domain, parent domain and interaction domain and has shown excellent validity and reliability [30]. In the present sample, internal consistency was good for the child (α = 0.84) and parent (α = 0.87) domains and somewhat lower internal consistency for the interaction domain (α = 0.65), consistent with the small number of items in this domain.

#### Clinician-rated functioning and severity

Global functioning was assessed using the Global Assessment of Functioning (GAF; [31]), a 1–100 scale reflecting overall psychological, social, and occupational functioning. Illness severity was rated using the Clinical Global Impressions–Severity scale (CGI-S; [32]), a 7-point clinician-rated measure based on symptom severity and associated functional impairment.

#### Family/friend history

Family and/or friend history of self-injurious behaviour was assessed using four dichotomous (yes/no) self-developed items asking adolescents whether a family member had engaged in NSSI, whether a family member had died by suicide, whether a close friend had engaged in NSSI, and whether a close friend had died by suicide. One point was assigned for each affirmative response, yielding a sum score ranging from 0 to 4, with higher scores indicating greater exposure to NSSI and suicide attempts among relatives and close friends.

### Safety planning

At T3, an individualized safety plan was developed collaboratively with the adolescent and, where appropriate, their parents or guardians, based on the Safety Planning Intervention framework described by Stanley and Brown [15]. The safety plan comprised identification of personal warning signs, internal coping strategies, social contacts for distraction and support, professional emergency contacts, and strategies to reduce access to potentially lethal means. The plan was documented in written form and provided to the adolescent and parents or guardians to facilitate implementation.

### Ethical considerations

The authors assert that all procedures contributing to this work comply with the ethical standards of the relevant national and institutional committees on human experimentation and with the Helsinki Declaration of 1975, as revised in 2013. All procedures involving human participants were approved by the local ethics committee of the University of Regensburg (approval number: 19-1426-101). The study was registered in the German Clinical Trials Register (DRKS; DRKS00017586**).**

### Ethics approval and consent to participate

Written informed consent was obtained from all participants and their parents or legal guardians prior to inclusion in the study. Participants were informed that their data would be anonymized and used for research purposes, and that participation was voluntary and could be withdrawn at any time without consequences for their clinical care.

### Statistical analyses

Changes in primary outcomes (NSSI behaviour and thoughts; suicidal thoughts and attempts; intensity of thoughts; motivation; emotional reactivity; psychological distress) across T2, FU1, and FU2 were examined using Friedman tests. Longitudinal analyses included all participants meeting inclusion criteria at baseline. Individuals with persistent zero values across all time points were retained to reflect the full clinical cohort. In addition to frequency-based analyses, changes in the prevalence of each behaviour were examined by dichotomising the past-month variables into any occurrence versus none and comparing these across T2, FU1, and FU2 using Cochran’s Q tests. This allowed frequency (number of occurrences) and prevalence (proportion of participants reporting any occurrence) to be evaluated separately, as the two may diverge when a subgroup continues a behaviour at high frequency while others cease. Percentages reported in the change-over-time analyses are based on participants with data at all three assessment points and therefore differ slightly from the full-sample percentages reported in Table 1, which describe all participants available at each timepoint.

Associations between outcomes and candidate predictors (general psychological distress, patient motivation, parental motivation, and parental stress, family/friend history) were explored using Kendall’s τ correlations with false discovery rate correction for multiple testing [33]. Group differences related to perceived benefit of the safety plan were examined using Mann-Whitney *U* and Kruskal-Wallis tests.

Longitudinal associations between predictors and outcomes were examined using linear mixed-effects models (LMEs), including time as a fixed effect and participant as a random effect. Random-effects structures were determined by model comparison using likelihood ratio tests. Separate models were computed for NSSI behaviour, NSSI thoughts, and suicidal thoughts; suicide attempts were not modelled due to insufficient variance. Candidate predictors were selected based on theoretical relevance and bivariate associations. LMEs were chosen because they allow examination of relationships across all time points while accounting for participant-specific variance and handling missing data efficiently [34].

The general modelling strategy for the three outcome variables was the following:

1. *NSSI*_*Behavior~*_*Motivation*_*Patient*_*+ ERS*_*Sensitivity*_ *+ GSI + EBI*_*Parent*_ *+ Intensity*_*NSSI*_ *+ SafetyPlan*_*Parent*_ *+ Time + (1|Participant)*
2. *Suicidal*_*Thoughts*_ *~ Age + Motivation*_*Patient*_ *+ ERS*_*Total*_ *+ Intensity*_*NSSI*_ *+ GSI + Time + (1|Participant)*
3. *NSSI*_*Thoughts*_*~ Motivation*_*Patient*_ *+ ERS*_*Total*_ *+ GSI + Intensity*_*NSSI*_ *+ Family/Friend History + Time + (Time|Participant)*

Here, *Motivation*_*Patient*_ refers to the PMC score, *EBI*_*Parent, Child, Interaction*_ refer to the three domains of parental stress, *Intensity*_*NSSI*_ is the intensity of NSSI thoughts at the worst time point. *ERS* refers to either the sensitivity scale or the total score. Random intercepts were specified as *(1|Participant)*, and random slopes as *(Time|Participant).* For NSSI thoughts, the random slope model performed better than the random intercept model, whereas for suicidal thoughts and NSSI behaviour the random intercept model was retained.

Model fitting followed best practice recommendations for linear mixed-effects modelling according to Barr et al. [35]. All mixed-effects models were computed using R (version 4.0.2; [36]) with the *lme4* and *lmerTest* packages. All other statistical analyses were conducted using IBM SPSS Statistics for Windows, version 29.0 [37]. The significance level was set at *α* = 0.05. Effect sizes were interpreted using conventional benchmarks (small ≈ 0.10/0.01, moderate ≈ 0.30/0.06, large ≈ 0.50/0.14, depending on the metric [38]). For linear mixed-effects models, semi-partial *R*² values were reported. Sample sizes varied across analyses due to missing data and outcome-specific inclusion criteria.

## Results

### Sample characteristics

An overview of sociodemographic and clinical sample characteristics is depicted in Table 1. Overall, *N* = 105 children and adolescents took part in the study between July 2019 and June 2021, mean age = 14.86 (*SD* = 1.57, range = 11–18). At FU2, a total of *n* = 71 participants remained. Reasons for non-participation included relocation, transfer to treatment elsewhere and failure to attend the scheduled follow-up appointments. Additional participants were recruited but had to be excluded due to language difficulties (*n* = 2), terminating their participation (*n* = 4) or exhibiting neither suicidal ideation nor SITB (*n* = 9). There were no significant differences between boys and girls in any of the relevant variables with the exception of the ERS at FU2 (*z* = −2.26, *r* = −.27, *p* = .024) and the GSI at all time points (*z* = − 2.47 – −3.15, *r* = .29–0.37, *p* = .002 − .013). Of the 105 participants assessed at T2, 78 (74.3%) took part in FU1 and 71 (67.6%) in FU2. Completers (*n* = 71) and non-completers (*n* = 34) were compared on baseline variables using Mann-Whitney U tests. The groups did not differ significantly in age, past-month frequency of suicidal thoughts, suicide attempts, NSSI thoughts or NSSI behaviour, illness severity (CGI) or global functioning (GAF) (all *p* > .30, all *r* ≤ .10).

**Table 1:** Sociodemographic and clinical characteristics of participants over three time points

		Time Points					
		T2 (N = 105)		FU1 (N = 78)		FU2 (N = 71)	
		M (SD)		M (SD)		M (SD)	
**Age**		14.86 (1.57)		14.87 (1.60)		14.85 (1.64)	
		**N**	**%**	**N**	**%**	**N**	**%**
**Gender**							
Female		80	76.2	59	75.6	53	74.6
Male		24	22.9	19	24.4	18	25.4
Missing		1	0.9				
**School type** ^**1**^							
Mittelschule		24	22.9	15	19.2	14	19.7
Realschule		27	25.7	24	30.8	22	31.0
Gymnasium		26	24.8	21	26.9	20	28.3
Förderschule		2	1.9	0	0.0	0	0.0
Erziehungshilfe-Schule		1	1.0	0	0.0	0	0.0
Berufsschule		12	11.3	11	14.1	11	15.3
No school		1	1.0	0	0.0	0	0.0
Other school		6	5.7	4	5.2	3	4.3
Missing		6	5.7	3	3.8	1	1.4
		***N*** **, M (SD)**		***N*** **, M (SD)**		***N*** **, M (SD)**	
**Family/friend history** ^**2**^		N = 104, 0.90 (0.87)		N = 77, 0.88 (0.90)		N = 71, 0.87 (0.91)	
		***N*** **, M (SD)**		***N*** **, M (SD)**		***N*** **, M (SD)**	
**Suicidal thoughts**							
Lifetime prevalence		92.4​%		96.2​%		97.1​%	
Last month prevalence		79.2​%		64.1​%		52.2​%	
Last month frequency		N = 101, 2.92 (5.99)		N = 78, 3.12 (5.94)		N = 71, 2.55 (5.90)	
**Suicide attempts**							
Lifetime prevalence		35.9		42.3		43.7	
Last month prevalence		24.3​%		9.0​%		4.2​%	
Last month frequency		N = 103, 0.34 (1.06)		N = 78, 0.10 (0.35)		N = 71, 0.07 (0.35)	
**NSSI thoughts**							
Lifetime prevalence		87.5		88.5		88.7	
Last month prevalence		73.3​%		60.1​%		57.7​%	
Last month frequency		N = 101, 3.89 (7.68)		N = 78, 4.40 (8.22)		N = 71, 2.96 (5.54)	
**NSSI behaviour**							
Lifetime prevalence		84.6		87		87.3	
Last month prevalence		61.0​%		54.5​%		47.9​%	
Last month frequency		N = 100, 5.19 (8.18)		N = 77, 5.21 (9.15)		N = 71, 3.44 (8.95)	
		***N*** **, M (SD)**					
**EBI (child domain)**		N = 97, 5.63 (1.48)					
**EBI (parent domain)**		N = 97, 5.20 (1.50)					
**EBI (interaction)**		N = 97, 6.22 (1.23)					
				**N**	**%**	**N**	**%**
**Safety plan (parents)**				71	100	67	100
Helpful				33	46.5	27	40.3
Not helpful				5	7.0	5	7.5
Not assessable				33	46.5	35	52.2
**Safety plan (patients)**				78	100	71	100
Helpful				28	35.9	21	29.6
Not helpful				16	20.5	24	33.8
Not assessable				34	43.6	26	36.6

Note. For each behaviour, lifetime prevalence is reported as the percentage of the available sample; last-month data are reported as the percentage of the available sample reporting the behaviour at least once in the past month (prevalence), and the mean number of occurrences in the past month (frequency). Lifetime prevalence is computed within the sample available at each timepoint; differences across timepoints reflect changes in sample composition due to attrition rather than new onset. NSSI = Non-suicidal self-injury; EBI = Parenting Stress Inventory; T2 = structured clinical appointment, FU1 = first follow up, FU2 = second follow up

^1^School types in Germany: Mittelschule (9 years of elementary school), Realschule (intermediate secondary school, 4 years of elementary school and 6 years of secondary school), Gymnasium (higher level education, 4 years of elementary school and 8-9 years of secondary school), Berufsschule (2 to 3 years of vocational training school most commonly after Mittelschule or Realschule), Förderschule (special needs school), Erziehungshilfe-Schule (school for educational assistance age 6 till graduation)

^2^Family/friend history = suicide attempt by a family member, suicide attempt by a close friend, NSSI behaviour by a family member, NSSI behaviour by a close friend; for each risk factor 1 point was added with a range of 0-4

Additionally, family/friend history was examined for its association with NSSI thoughts, NSSI behaviour, and suicidal thoughts across time using Kendall’s τ with FDR correction. Family/friend history showed a significant and strengthening association with NSSI thoughts across all assessment points (T2: τ = 0.20; FU1: τ = 0.22; FU2: τ = 0.34; all q < 0.05), and a significant association with NSSI behaviour at FU2 (τ = 0.25, q < 0.05). No significant associations with suicidal thoughts were observed after correction.

### Changes over time

**Fig. 2:**
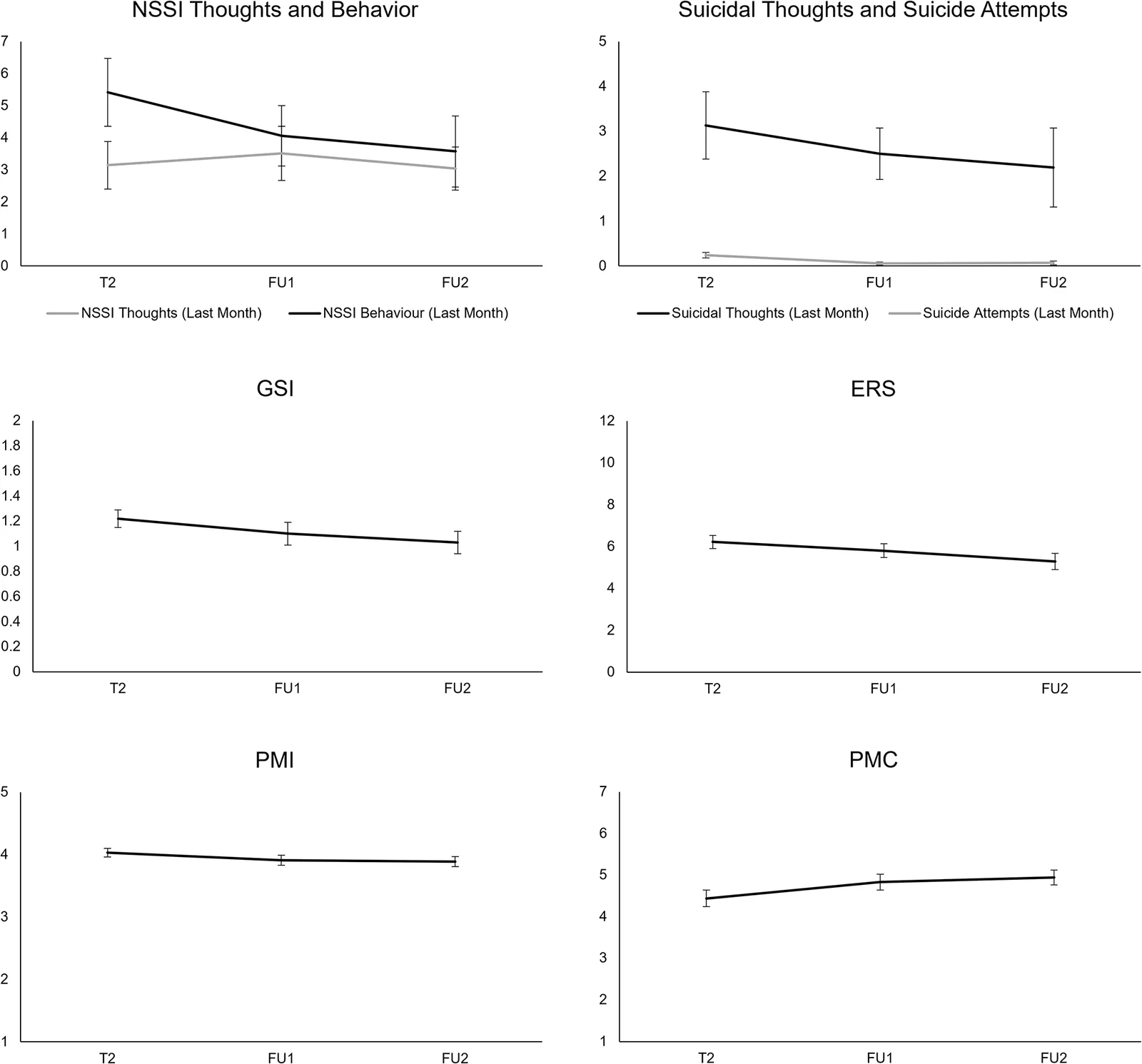
Overview of frequency of key variables over time. Note. NSSI = Non-Suicidal Self-Injury; GSI = Global Severity Index; ERS = Emotional Reactivity Scale; PMI = Parental Motivation Index; PMC = Patient Motivation for Change. The y-axis refers to frequency scores and the respective questionnaire scores

The variables NSSI behaviour, NSSI thoughts, suicidal thoughts, suicide attempts, intensity of NSSI/suicidal thoughts, PMI, PMC, and ERS were examined for changes over time (T2, FU1, FU2). Figure 2 depicts changes over time based exclusively on participants who provided data at all three assessment points (T2, FU1, and FU2).

Changes over time were examined both for frequency, using Friedman tests on past-month counts, and for prevalence, using Cochran’s Q on the proportion reporting any occurrence in the past month, among participants with data at all three assessment points.

For suicide attempts, both frequency (χ2(2) = 16.53, W = 0.12, *p* < .001) and prevalence decreased significantly (Q = 16.53, *p* < .001, *N* = 70), with the proportion reporting any attempt in the past month falling from 22.9% at T2 to 4.3% at FU2. For NSSI behaviour, frequency decreased (χ2(2) = 5.73, W = 0.05, *p* = .048), whereas the decline in prevalence did not reach significance (Q = 2.74, *p* = .254, *N* = 68; 58.8% to 48.5%). The opposite pattern emerged for thoughts: neither NSSI thoughts (χ2(2) = 0.38, W = 0.00, *p* = .829) nor suicidal thoughts (χ2(2) = 5.35, W = 0.04, *p* = .069) changed significantly in frequency, yet the prevalence of both declined significantly (NSSI thoughts: Q = 11.00, *p* = .004, *N* = 69, from 75.4% to 59.4%; suicidal thoughts: Q = 17.87, *p* < .001, *N* = 68, from 79.4% to 51.5%). Intensity of thoughts remained unchanged over time (χ2(2) = 0.30–3.53, W ≤ 0.03, *p* = .171-0.860).

Parental motivation decreased over time (χ^2^(2) = 6.41, *W* = 0.05, *p* = .041), whereas patient motivation increased (χ^2^(2) = 9.02, *W* = 0.06, *p* = .011). Finally, both the GSI and the ERS score significantly improved over time (GSI: χ^2^(2) = 10.86, *W* = 0.08, *p* = .004; ERS: χ^2^(2) = 9.55, *W* = 0.07, *p* = .008).

Among clinician-rated outcomes assessed at T2 and FU2, CGI decreased (*z* = − 2.67, *p* = .008, *r* = − .71) and GAF increased (*z* = − 5.34, *p* < .001, *r* = − .65).

### Benefit of the safety plan

Parental assessment of the safety plan at FU2 was significantly associated with NSSI behaviour at FU2 (*χ²*= 9.22, ε² = 0.13, *p* = .010), with greater reductions observed when parents rated the safety plan as helpful. There were no differences in NSSI behaviour/thoughts or suicidal thoughts, the GSI, the ERS, patient or parent motivation. Regarding adolescents’ assessment of the safety plan, no associations were identified (*p* > .05). The association between assessment of the safety plan and suicide attempts was not examined, as the low frequency of attempts at follow-up (e.g. 3 of 71 at FU2) precluded meaningful analysis.

### Correlations between main variables

Following FDR correction within outcome families, several variables remained significantly associated with suicidal thoughts, including age (τ = 0.20, *p* = .003), emotional reactivity (τ = 0.28, *p* = .001), general psychological distress (τ = 0.25, *p* = .004), patient motivation (τ = −0.28, *p* = .002), and intensity of NSSI thoughts (τ = 0.38, *p* < .001). For NSSI thoughts, significant associations were observed with emotional reactivity (τ = 0.29, *p* = .002), general psychological distress (τ = 0.25, *p* = .005), patient motivation (τ = −0.36, *p* < .001), and intensity of NSSI thoughts (τ = 0.34, *p* = .002). In contrast, NSSI behaviour was significantly associated only with patient motivation (τ = −0.35, *p* < .001) and intensity of NSSI thoughts (τ = 0.29, *p* = .010). No other correlations remained significant after correction.

### Linear mixed-effects models predicting NSSI behaviour and thoughts

The maximized model for suicidal thoughts did not provide a significantly better fit than the null model and is therefore not reported in detail. The model for NSSI behaviour and NSSI thoughts performed significantly better than the null model (see Table S1 in the supplement). For NSSI behaviour, PMC emerged as the only significant predictor of NSSI behaviour over time, whereas no predictors were significant for NSSI thoughts (see Table 2).

**Table 2:** Linear mixed effect model predicting NSSI thoughts and NSSI behaviour with fixed effects

Outcome Variable	Fixed Effect	Estimate	SD	t-value	*p*-value	$\:{\boldsymbol{R}}_{\boldsymbol{\beta\:}\boldsymbol{*}}^{2}$	$\:{\boldsymbol{R}}_{\boldsymbol{\beta\:}\boldsymbol{*}}^{2}\:\boldsymbol{C}\boldsymbol{I}$
NSSI Thoughts	PMC	-1.03	0.57	-1.83	0.070	0.01	0.00 - 0.05
	ERS: Total	-0.51	0.36	-1.45	0.150	0.01	0.00 - 0.04
	GSI	2.21	1.48	1.49	0.138	0.01	0.00 - 0.04
	Intensity: NSSI	1.58	1.06	1.49	0.138	0.01	0.00 - 0.04
	Family History	0.91	0.86	1.05	0.297	0.00	0.00 - 0.03
	Time	-1.22	1.25	-0.97	0.332	0.01	0.00 - 0.04
NSSI Behaviour	**PMC**	**-1.64**	**0.53**	**-3.07**	**0.003**	**0.05**	**0.01 - 0.13**
	ERS: Sensitivity	1.02	1.11	0.93	0.356	0.01	0.00 - 0.05
	GSI	-0.09	1.50	-0.06	0.952	0.00	0.00 - 0.03
	EBI: Parent Domain	-0.89	0.61	-1.46	0.150	0.02	0.00 - 0.08
	Intensity: NSSI	-1.43	1.07	-1.34	0.181	0.01	0.00 - 0.06
	Safety Plan	1.10	1.29	0.85	0.399	0.01	0.00 - 0.06
	Time	-0.35	0.61	-0.57	0.572	0.00	0.00 - 0.03

Note. For individual model terms, the semi-partial (marginal) R^2^ (${{R}}_{{\beta\:}{*}}^{2}$) is reported including confidence intervals. NSSI = Non-suicidal self-injury; PMC = Patient Motivation for Change; ERS = Emotion Reactivity Scale; GSI = Global Severity Index

## Discussion

This study examined longitudinal changes in NSSI behaviour, NSSI thoughts, suicidal ideation, and suicide attempts following standardized emergency management in adolescents. The central finding concerned a dissociation between the frequency and the prevalence of self-injurious thoughts and behaviours. Suicide attempts declined on both measures, though from a low base rate. For NSSI behaviour, frequency decreased while prevalence declined only modestly, whereas for NSSI thoughts and suicidal ideation the reverse held: prevalence, the proportion of adolescents reporting any such thoughts in the last month, decreased significantly, while their overall frequency did not change. In other words, change in ideation was concentrated at the level of prevalence rather than frequency. This pattern is increasingly recognized in longitudinal research and suggests that behavioural and cognitive components of SITB may show partially dissociable courses over time, particularly in clinically referred samples [14].

These behavioural improvements were accompanied by reductions in clinician-rated illness severity and increases in global functioning, further supporting the interpretation of short-term clinical stabilization.

Large-scale trajectory and cross-lagged studies consistently demonstrate that SITB and suicidal ideation are dynamically intertwined over time, yet not redundant [11, 12]. Against this background, the present findings extend prior work by demonstrating that behavioural improvement following emergency intervention may occur alongside reductions in the prevalence of ideation but not its frequency, even as overall emotional distress improves.

### Behaviour–ideation dissociation and clinical risk

The present dissociation between behavioural and ideational change is consistent with evidence from high-risk clinical samples. In adolescents with mood disorders, both remitted and persistent NSSI groups continue to report elevated suicidal ideation compared to peers that never engaged in NSSI, while only persistent NSSI predicts ongoing suicidal behaviour [14]. Similarly, longitudinal inpatient and outpatient cohorts show substantial reductions in NSSI frequency over time but persistent ideational distress in a sizeable subgroup [39, 40]. The present study partly aligns with these findings but adds nuance: while behavioural risk decreased following emergency management with intervention, ideational change was expressed as a reduction in prevalence rather than frequency, with fewer adolescents reporting suicidal or NSSI-related thoughts while their overall frequency did not change.

Importantly, although the overall frequency of ideation did not change, cessation of NSSI behaviour is clinically meaningful. Longitudinal and registry-based studies demonstrate that NSSI independently predicts later suicidal ideation, suicide attempts, psychiatric morbidity, and functional impairment, even after controlling for baseline psychopathology [41–43]. Thus, behavioural reduction should be considered a critical early target in emergency care, even when ideation persists.

### Emotion reactivity and global psychological distress

Consistent with prior emergency and clinical cohort studies, emotional reactivity and general psychological distress improved over time in the present sample. Emotional reactivity has been identified as both a predictor and consequence of NSSI engagement, particularly in adolescents presenting for emergency care [17]. Longitudinal research further suggests that emotion dysregulation is closely linked to the persistence of NSSI, whereas depressive symptoms and hopelessness are more strongly associated with suicidal ideation and attempts [44–46]. This distinction may help explain the observed dissociation in our study, with behavioural improvements occurring despite the relative stability of ideation frequency.

The lack of predictive effects of emotional reactivity and global distress on ideational outcomes in the present mixed-effects models may reflect the relative stability of ideation frequency in high-risk adolescents, as well as the limited time frame of follow-up. Prior work suggests that ideation trajectories are often more chronic and less responsive to short-term improvements in emotional functioning than behavioural outcomes [47, 48]. It is also conceivable that cognitive reappraisal and shifts in suicidal ideation emerge only after sustained behavioural change over a longer period, which may not have been captured within the present follow-up interval. Similarly, although family and/or friend history of NSSI and suicide attempts was bivariately associated with NSSI thoughts, it did not independently predict this outcome in the mixed-effects model once emotional reactivity, distress, and symptom intensity were accounted for, suggesting its association is not independent of broader emotional vulnerability.

### Motivation and parental involvement

Adolescent motivation for change emerged as the sole significant predictor of reductions in NSSI behaviour over time. This finding is notable given the few longitudinal studies directly measuring readiness or motivation to stop NSSI. In a pilot randomized trial, safety planning combined with motivational interviewing increased adolescents’ confidence in their ability to refrain from suicidal behaviour and enhanced their engagement with coping strategies and use of the safety plan [49], suggesting motivation may be a meaningful treatment target. Daily monitoring studies further indicate that engagement with safety plans varies by sex and is not simply a function of ideation intensity, highlighting the role of motivational and contextual factors [50].

In the present study, parental perception of the safety plan, but not adolescents’ own ratings, was associated with reduced NSSI behaviour at follow-up. Notably, parental motivation for treatment decreased slightly over time, suggesting that parent engagement may attenuate once acute crisis management has stabilized. This underscores the importance of actively maintaining parental involvement beyond the initial emergency phase. This finding converges with longitudinal evidence that family cohesion, parental involvement, and emotion socialization predict better NSSI outcomes, while family conflict and parental psychological control are associated with more persistent risk trajectories [51–53]. Together, these findings underscore the importance of parent engagement in emergency interventions aimed at behavioural risk reduction.

### Safety planning

In the present study, parental perception of the safety plan was associated with reductions in NSSI behaviour, whereas no effects were observed for NSSI thoughts or suicidal ideation. Safety planning is widely implemented as a brief intervention in emergency settings, yet its specific effects on adolescent NSSI and suicidal outcomes remain incompletely understood. Existing studies primarily demonstrate feasibility, acceptability, and improvements in ideational and motivational outcomes rather than consistent reductions in self-injurious behaviour or suicide attempts [49, 50, 54]. Against this background, the present findings suggest that safety planning may support short-term behavioural stabilization, particularly when parents are engaged, but appears insufficient to reduce the frequency of persistent ideational symptoms on its own. This supports conceptualizing safety planning as an initial containment strategy rather than a standalone treatment for persistent suicidal ideation or NSSI thoughts.

### Clinical implications

Taken together, the findings support a stepped-care model in emergency child and adolescent psychiatry. Early interventions should prioritize behavioural risk reduction, foster adolescent motivation for change, and actively involve parents or guardians. However, persistent NSSI thoughts and suicidal ideation (frequency) warrant continued monitoring and targeted follow-up interventions focused on emotional regulation, cognitive processes, and interpersonal stressors. Failure to address ideation separately may leave adolescents vulnerable despite apparent behavioural improvement.

### Limitations

Several limitations should be acknowledged. The observational design precludes causal inference regarding intervention effects. In particular, the study did not include a control or comparison group. All analyses examined change over time within a single group of adolescents who received the standardized emergency management, so we cannot determine whether the observed improvements exceed those that would occur after acute crisis care alone. Because participants were recruited at the point of acute crisis, some of the observed improvement may also reflect regression to the mean or the natural course of the crisis. Future studies should compare standardized emergency management with acute emergency care as usual, ideally in a randomized controlled design, to establish its specific effects. The relatively short follow-up intervals may have limited the detection of longer-term changes, particularly in ideational outcomes; future studies should include extended follow-up periods to clarify the stability of observed effects. Sample size and attrition may have reduced statistical power to detect predictors of change in suicidal ideation. Approximately one third of participants did not complete FU2. Although completers and non-completers did not differ in baseline clinical characteristics, selective dropout based on unmeasured factors cannot be ruled out. Some measures relied on self-report, and the adapted motivation measure, although internally consistent, requires further external validation. Furthermore, findings derive from a single outpatient emergency service and may not generalize to other clinical settings and, because adolescents requiring prolonged inpatient admission were treated separately and not included, findings apply to adolescents whose acute crisis was manageable on an outpatient basis rather than to all emergency presentations. Finally, although all participants received treatment as usual and clinical recommendations were issued following emergency management, we did not systematically record which recommendations or additional treatments individual adolescents took up during the follow-up period. Any additional treatment received during this time may therefore have contributed to the observed changes and cannot be ruled out.

Nevertheless, strengths of the study include the prospective multi-informant design, the standardized emergency management protocol, and the repeated assessment of both behavioural and ideational SITB, allowing examination of their potentially dissociable trajectories.

### Summary

In line with contemporary longitudinal and trajectory-based research, this study demonstrates that behavioural and ideational components of self-injury and suicidality improve along different dimensions following emergency intervention. Suicide attempts declined on both frequency and prevalence, though from a low base rate; NSSI behaviour declined mainly in frequency; and suicidal and NSSI-related thoughts declined mainly in prevalence, with fewer adolescents reporting them but no reduction in their overall frequency. Motivation and parental engagement appear central to behavioural change, whereas ideation improved less completely and was not predicted by the measured variables. These findings reinforce the need to conceptualize self-injurious and suicidal thoughts and behaviours as distinct but interacting therapeutic targets in adolescent emergency care.

## Supplementary Information

Below is the link to the electronic supplementary material.

Supplementary Material 1: Table S1

## Data Availability

The data that support the findings of this study are available from the corresponding author upon reasonable request. The data are not publicly available due to ethical restrictions and the sensitive nature of information that could compromise the privacy of research participants.
