# Supplementary material for: Longitudinal trajectories of non-suicidal self-injury, suicidal ideation, and suicide attempts following standardized outpatient emergency management in adolescents
Source: Child Adolesc Psychiatry Ment Health. 2026 Nov 6;20:155. doi: 10.1186/s13034-026-01204-3 (PMC13749642; doi:10.1186/s13034-026-01204-3)
Supplement: Supplementary file 1 [file 13034_2026_1204_MOESM1_ESM.docx]

| **Table S1** *Model comparisons of linear-mixed effect models predicting suicidal thoughts, NSSI thoughts and NSSI behaviour.* | | | | | | | |  |
| --- | --- | --- | --- | --- | --- | --- | --- | --- |
| Outcome Variable | Model | AIC | BIC | logLik | Vs. Null Model | | |  |
|  |  |  |  |  | *Χ*^2^ | *p*-value | |  |
| Suicidal Thoughts | Null Model (Intercept) | 1416.60 | 1426.80 | -705.32 |  |  | |  |
|  | Null Model (Slope) | 1414.80 | 1431.70 | -702.37 | 5.88 | .053 | |  |
|  | Max Model | 1418.20 | 1455.40 | -689.08 | 14.47 | .070 | |  |
| NSSI Thoughts | Null Model (Intercept) | 1842.20 | 1852.40 | -918.09 |  |  |  |  |
|  | Null Model (Slope) | 1766.80 | 1783.80 | -878.40 | 79.36 | <.001 | |  |
|  | **Max Model** | **1762.40** | **1799.80** | **-870.18** | **16.45** | **.012** | |  |
| NSSI Behaviour | Null Model (Intercept) | 1271.40 | 1280.90 | -632.68 |  |  | |  |
|  | Null Model (Slope) | 1273.90 | 1289.80 | -631.95 | 1.46 | .481 | |  |
|  | **Max Model** | **1267.90** | **1302.90** | **-622.96** | **19.45** | **.013** | |  |
| *Note.* χ² and p-values refer to likelihood-ratio tests comparing nested models (Null Model with intercept vs. Null Model with slope; Null Model with slope vs. Max Model). NSSI = Non-suicidal self-injury; AIC = Akaike Information Criterion; BIC = Bayesian Information Criterion; logLik = Log-Likelihood. | | | | | | | |  |
